# The relationship between hospital and ehr vendor market dynamics on health information organization presence and participation

**DOI:** 10.1186/s12911-018-0605-y

**Published:** 2018-05-08

**Authors:** Sunny C. Lin, Julia Adler-Milstein

**Affiliations:** 1 Department of Health Management and Policy, School of Public Health, University of Michigan, Ann Arbor, MI USA; 2 Department of Medicine, Center for Clinical Informatics and Improvement Research, University of California, San Francisco, CA USA

**Keywords:** Health information exchange, Electronic health records, Systems integration

## Abstract

**Background:**

Health Information Organizations (HIOs) are third party organizations that facilitate electronic health information exchange (HIE) between providers in a geographic area. Despite benefits from HIE, HIOs have struggled to form and subsequently gain broad provider participation. We sought to assess whether market-level hospital and EHR vendor dynamics are associated with presence and level of hospital participation in HIOs.

**Methods:**

2014 data on 4523 hospitals and their EHR vendors were aggregated to the market level. We used multivariate OLS regression to analyze the relationship between hospital and vendor dynamics and (1) probability of HIO presence and (2) percent of hospitals participating in an HIO.

**Results:**

298 of 469 markets (64%) had HIO presence, and in those markets, 47% of hospitals participated in an HIO on average. In multivariate analysis, four characteristics were associated with HIO presence. Markets with more hospitals, markets with more EHR vendors, and markets with an EHR vendor-led HIE approach were *more likely* to have an HIO. Compared to markets with low hospital competition, markets with high hospital competition had a 25 percentage point *lower probability* of HIO presence. Two characteristics were associated with level of hospital HIO participation. Markets with more hospitals as well as markets with high vendor competition (compared to low competition) had lower participation.

**Conclusion:**

Both hospital and EHR vendor dynamics are associated with whether a market has an HIO as well as the level of hospital participation in HIOs.

**Electronic supplementary material:**

The online version of this article (10.1186/s12911-018-0605-y) contains supplementary material, which is available to authorized users.

## Background

Fragmented healthcare delivery has resulted in silos of health information and the challenge of ensuring that patient information is shared between providers. Information sharing is vital to care coordination, and when done electronically, can be more comprehensive, accurate, and timely, leading to a reduction in redundant testing, improved patient safety, and better quality of care [1, 2]. Health Information Organizations (HIOs) are third party organizations that provide the governance and technical infrastructure to enable electronic health information exchange (HIE) between providers in a geographic area. As opposed to other HIE approaches that place restrictions on who can participate in sharing data, such as Enterprise Health Information Exchanges or EHR Vendor Exchanges, HIOs promote community-wide HIE participation [3] and are a community approach to HIE that may be less likely to lead to information blocking (the intentional and unreasonable interference with electronic exchange of health information) [4].

Although HIOs represent an important option for community-level connectivity, HIOs have struggled to identify sustainable business models, which may be driving the observed decline in the number of HIOs [5] and leads to uncertainty about their viability. Critical to HIO sustainability is whether hospitals perceive participation as valuable, which is influenced both by hospital dynamics and by how EHR vendors work with HIOs. Hospitals have expressed concern about the potential loss of patients and revenue that could result from sharing data with competitors through HIOs, particularly if participation costs are high. Anecdotal reports of EHR vendors charging prohibitive fees or withdrawing support for connectivity have raised concerns about the influence of EHR vendors on the sustainability of HIOs, especially as EHR vendors are increasingly offering their own HIE networks [6].

While prior studies on HIOs have examined what predicts whether or not hospitals participate in an HIO [7], it is not yet known whether market conditions, particularly those related to dynamics of hospitals and EHR vendors, are associated with HIO presence and the level of hospital participation. This question has important implications for understanding the future of HIOs and the effectiveness of policies designed to promote their sustainability. In particular, many HITECH policies seeking to increase HIE have targeted individual organizations. To the extent that market characteristics predict HIO presence and level of hospital participation, it may be more effective to pursue policies at the market level.

In this study, we sought to understand how hospital and EHR vendor market dynamics are related to HIO presence and level of hospital participation in HIOs. Market dynamics may hinder **HIO presence** if an HIO is unable to negotiate tensions between competing hospitals or vendors. Even in markets that have successfully established an HIO, these same market dynamics may limit**the level of hospital participation in HIOs**. We answer the following questions: (1) Do market-level hospital and EHR vendor characteristics differ in markets with and without an HIO? (2) In markets with an HIO, are market-level characteristics associated with the level of hospital participation in HIOs?

## Methods

### Data

Data on US hospitals (excluding mental, children’s, and federal government hospitals) from the 2014 AHA Survey and 2014 AHA IT Supplement were aggregated to the Health Service Area (HSA) and merged with data from the 2012 Dartmouth Atlas and the 2014 Area Health Resource File. When 2014 AHA data was missing, we used the most recently available data from the 2008–2013 American Hospital Association (AHA) Surveys and 2012–2013 AHA IT Supplements. Because many HSAs contain a single hospital, areas bound by HSAs may not adequately capture interactions between hospitals. We therefore combined HSAs within Health Referral Regions (HRRs, larger geographic areas created by aggregating HSAs based on patient referrals for tertiary medical care) based on whether or not there was at least one HIO in the HSA. This effectively split HRRs into two areas, one that contains all HSAs in the HRR that have HIO presence, and one that contains all HSAs in the HRR that do not have HIO presence. Each of these areas was considered an individual market.

Markets were dropped if there were not enough hospitals in the market that responded to the AHA IT Supplement. If there were at least 7 hospitals in the market, at least one-third of the hospitals in the market had to have responded to the IT supplement to remain in the data set. If there were fewer than 7 hospitals in the market, at least 3 of the hospitals had to have responded to remain in the data set. Additional markets were dropped if no hospitals had data available for the following revenue-related measures: percent of inpatient days from Medicare, percent of inpatient days from Medicaid, and percent of revenue from shared risk or capitated payment sources. A total of 72 markets were dropped (63 missing IT data and 9 missing hospital revenue-related data) resulting in a final analytic data set of 469 markets, representing 2648 HSAs and 4523 hospitals (Table 1).

**Table 1:** Characteristics of markets by HIO presence and level of hospital participation

Variables	All Areas	No HIO Presence	HIO Presence		Hospital Participation	
Number of Markets	469	171	298		298	
*Categorical Variables*	*n(%)*	*n(%)*	*n(%)*	*P-value*	Mean (SD)	*P-value*
Number of Hospitals				< 0.001		< 0.001
Low (1–4)	175 (37%)	106 (62%)	69 (23%)		58% (32)	
Moderate (5–8)	128 (27%)	36 (21%)	92 (31%)		41% (24)	
High (9+)	166 (35%)	29 (17%)	137 (46%)		47% (25)	
Total	469	171	198			
Hospital Competition (HHI)				< 0.001		0.33
Non-Competitive (0.46–1.00)	127 (27%)	67 (39%)	60 (20%)		59% (32)	
Moderately Competitive (0.25–0.45)	166 (35%)	62 (36%)	104 (35%)		45% (26)	
Highly Competitive (0.00–0.24)	176 (37%)	42 (25%)	134 (45%)		43% (20)	
Total	469	171	298			
For-Profit Hospital Marketshare				0.003		< 0.001
Low For-Profit Marketshare (0–57%)	357 (76%)	117 (68%)	240 (81%)		34% (34)	
High For-Profit Marketshare (> 57%)	112 (24%)	54 (32%)	58 (19%)		18% (18)	
Total	469	171	298			
No. of EHR Vendors				< 0.001		< 0.001
Low (1–2)	141 (30%)	21 (12%)	120 (40%)		55% (32)	
Moderate (3–4)	146 (31%)	45 (26%)	101 (34%)		44% (25)	
High (5+)	182 (39%)	105 (61%)	77 (26%)		44% (19)	
Total	469	171	298			
Vendor Competition (HHI)				< 0.001		0.35
Non-Competitive (0.63–1.00)	144 (31%)	76 (44%)	68 (23%)		57% (31)	
Moderately Competitive (0.38–0.62)	162 (35%)	57 (33%)	105 (35%)		46% (25)	
Highly Competitive (0.00–0.37)	163 (35%)	38 (22%)	125 (42%)		42% (20)	
Total	469	171	298			
Alternative HIE Approach (%)						
No (0–49% of hospitals on Epic)	256 (55%)	135 (79%)	121 (41%)	< 0.001	44% (27)	< 0.001
Yes (50–100% of hospitals on Epic)	213 (45%)	36 (21%)	177 (59%)		49% (24)	
Total	469	171	298			
*Continuous Variables*	*Mean (SD)*	*Mean (SD)*	*Mean (SD)*	*P-value*	*Coefficient (SE)*	*P-value*
Number of HSAs	6 (6)	5 (4)	6 (6)	0.018	0.16 (0.23)	0.46
% HIO Participation	30 (30)	0 (0)	47 (25)	< 0.001		
% Hospital Participation in Patient Centered Medical Home and/or Accountable Care Organizations	27 (27)	17 (26)	33 (25)	< 0.001	0.003 (0.001)	< 0.001
% Revenue Shared Risk Programs	1 (4)	1 (3)	2 (4)	0.062	0.12 (0.33)	0.72
% Inpatient Days Medicare	52 (21)	53 (15)	51 (9)	0.088	0.18 (0.16)	0.25
% Inpatient Days Medicaid	19 (9)	18 (10)	19 (8)	0.066	0.32 (0.19)	0.10
Hospital Beds per 1000 residents	24 (31)	15 (16)	29 (36)	< 0.001	−0.09 (0.04)	0.03
FTE Hospital Staff per 1000 residents	164 (197)	98 (91)	202 (229)	< 0.001	−0.01 (0.01)	0.12
Percentage of Hospitals in Urban Settings	62 (35)	46 (39)	72 (29)	< 0.001	−0.04 (0.05)	0.50
Number of Physicians (Weighted County Average)	409 (769)	260 (737)	495 (775)	0.001	0.00 (0.00)	0.65

*HIO* Health Information Organization,*HHI* Herfindahl-Hirschman Index, *EHR* Electronic Health Record, *HSA* Health Service Area, *FTE* Full Time Equivalent, *SD* Standard Deviation, *SE* Standard Error

Note: Percentages may not add up to 100% due to rounding; *p*-values based on ANOVA for categorical variables and linear regression for continuous variables

### Dependent variables

HIO presence in a market was measured using responses to a question from the AHA IT Supplement that asked about HIOs in a hospital’s area. An HSA was considered as having an HIO if at least one hospital in the HSA indicated that there was an HIO in their area, even if they did not participate. Level of hospital participation in HIOs was calculated using the percent of hospitals in the market that reported *actively participating* in an HIO on the AHA IT Supplement. Because HIO participation can, by definition, only occur in markets with an HIO, analysis of HIO participation was limited to markets with HIO presence.

### Independent variables

We selected six independent variables—three related to hospital dynamics and three related to vendor dynamics—that we hypothesized would be related to both the likelihood of HIO presence and the level of hospital HIO participation in markets with HIOs (Please see Additional file 1: Conceptual Model). We chose these variables based on those included in previous hospital-level studies that sought to predict which hospitals engage in HIE and adopt other types of health information technologies [8, 9].

#### Hospital market size

A greater number of hospitals may be associated with higher probability of HIO presence and a higher level of hospital HIO participation, because markets with more hospitals have more fragmented care and therefore see a greater need to establish an HIO and share information. Markets were categorized as having a low, moderate, or high number of hospitals using tertiles, which are cutoffs that split the sample into three equal parts and resulted in the following categories: low = 1–5 hospitals, moderate = 6–10, high = 11 + .

#### Hospital market competition

Hospital competition may be associated with lower probability of HIO presence and lower hospital participation because hospitals that are highly competitive may prefer more selective ways to exchange information, such as through Enterprise HIE or direct connections [3, 8, 10, 11]. We categorized markets as non-competitive, moderately competitive, and highly competitive using the Herfindahl-Hirschman index (HHI). The HHI is calculated using the sum of the squared market share of each hospital in the market. Market share was determined using the number of hospital beds in a hospital system. After calculating the HHI for the market, markets were categorized as non-competitive, moderately competitive, or highly competitive based on whether they were in top, middle, or bottom third of the distribution for HHI, respectively (HHI of 0.447–1 = noncompetitive, 0.239–0.439 = moderately competitive, 0–0.238 = highly competitive).

#### Hospital market ownership

For-profit hospitals may see HIO participation as a risky way to exchange information, since HIO participation makes valuable patient data more accessible and could reduce barriers for patients to receive care at competitors. Markets with a large for-profit market share may have lower probability of HIO presence and lower hospital participation. For-profit market share was calculated using the percent of beds owned by for-profit hospitals. Markets were considered as having high for-profit marketshare if they were in the top quartile of for-profit marketshare (> 57%).

#### EHR vendor market size

A higher number of EHR vendors that are used by hospitals in the market might increase the technical complexity and associated costs of connecting multiple EHR systems from different vendors [12–14]. This could improve the attractiveness of an HIO because each hospital would only need to establish a single connection to the HIO, increasing both the probability of HIO presence and the level of hospital participation. Alternatively, having more EHR vendors could increase the cost of HIO formation and hospital participation, decreasing the probability of HIO presence and reducing participation. The number of EHR vendors was calculated by counting the number of unique inpatient EHR vendors used by hospitals in the market. Markets were categorized as having a low, moderate, or high number of vendors using tertiles (low = 1–2 vendors, moderate = 3–4, high = 5+).

#### EHR vendor market competition

In competitive vendor markets, competing EHR vendors may be reluctant to support hospital participation in HIOs in an effort to maintain vendor lock-in [13, 15–17]. As a result, markets with competitive EHR vendors may have lower probability of HIO presence and lower hospital participation. Similar to hospital competition, vendor competition was determined using the Herfindahl-Hirschman Index of vendors in the market. Vendor market share was based on the number of hospital beds in hospitals that use the vendor’s inpatient EHR product. Markets were categorized as non-competitive, moderately competitive, and highly competitive based on whether they were in the top, middle, or bottom third of the distribution for vendor HHI (HHI of 0.592–1.000 = noncompetitive, 0.357–0.592 = moderately competitive, and 0–0.356 = highly competitive).

#### Market penetration of alternative (vendor-led) HIE approach

One specific EHR vendor - Epic - has a mature intra-Epic HIE in which the vast majority of Epic clients participate. Therefore, markets dominated by Epic have an alternative to HIOs and there may be a sufficient number of hospitals that prefer to exchange data through Epic’s HIE platform instead of an HIO, resulting in both a lower probability of HIO presence and lower hospital participation. Market penetration of an alternative (vendor-led) HIE approach was measured by calculating Epic marketshare using the percent of beds owned by hospitals whose primary inpatient vendor was Epic. A market was considered to have an alternative HIE approach if the Epic marketshare was greater than 50%.

We included seven market-level controls: percent of hospitals that participate in a Patient Centered Medical Home and/or Accountable Care Organization, average percent of revenue from alternative payment models (e.g. shared risk programs and capitated payments), average percent of inpatient days from Medicare/Medicaid, number of hospital beds per 1000 residents, number of full time hospital employees per 1000 residents, number of physicians per 1000 residents, and percent of hospitals in urban settings. These control variables were chosen because they may confound the relationship between hospital and EHR vendor market dynamics and HIO presence and hospital participation in HIOs. In markets where a higher percentage of hospital revenue is from shared risk programs, hospitals may be more likely to participate in an HIO effort to improve care coordination [12]. In markets where hospitals have a greater percentage of inpatient days paid for by Medicare and Medicaid, hospitals may be more inclined to participate in an HIO in order to meet Meaningful Use criteria [18]. Markets with a higher density of hospital beds, hospital staff, and physicians, as well as greater urbanicity, may have greater need for HIE to facilitate care coordination.

### Analysis

To answer our first research question about how hospital and vendor market characteristics differ for markets with and without an HIO, we conducted bivariate analyses on market characteristics and HIO presence, and then ran a multivariate linear probability model with state fixed effects. A linear probability model was chosen over the logistic model for ease of interpretation, such that coefficients can be interpreted as a change in the predicted probability of HIO presence given a unit change in the independent variable. However, as a robustness test, we ran a logistic regression model and the resulting odds ratios were compared with the linear probability model (Additional file 2: Logistic Regression Model for HIO Presence in Marekts with different Hospital and Vendor Characteristics).

To address our second research question about how characteristics of markets are associated with hospital participation, we conducted bivariate analyses on hospital participation and market characteristics using one-way ANOVAs for categorical variables and linear regression for continuous variables. We then ran a multivariate linear regression model with state fixed effects using hospital participation as the dependent variable for all markets with HIO presence.

In our dataset, the number of hospitals and number of vendors in the market were highly correlated (correlation coefficient = 0.83), while the number of hospitals and hospital competition, the number of vendors and vendor competition, and hospital and vendor competition were moderately correlated (correlation coefficients = 0.50, 0.74, 0.74 respectively). High multicollinearity may lead to two problems: (1) invalid interpretations of model coefficients as “the expected change in an outcome *holding all other variables constant”*, since, as a result of collinearity, a change in one variable necessitates a change in the collinear variable, and (2) high standard errors for collinear variables leading to insignificance. We addressed multicollinearity in three ways. First, in our primary models, we use tertiles of number of hospitals and vendors, and hospital and vendor competition instead of continuous variables. Second, as a robustness test, we ran our models using continuous variables, to ensure that the directionality of the estimated effect was consistent with our primary models. Finally, we ran our models and systematically excluded each of the collinear variables, and then compared standard errors and statistical significance to our primary models.

## Results

### HIO presence

In bivariate analyses, markets with and without an HIO differed significantly for all independent variables, but not always in the predicted direction: markets with an HIO had more hospitals (*p* < 0.001), had higher hospital competition (*p* < 0.001), were less likely to have high for-profit hospital marketshare (*p* = 0.003), had more EHR vendors (*p* < 0.001), had more competitive EHR vendors (p < 0.001) and were more likely to have an alternative HIE approach (i.e., be Epic dominant; *p* < 0.001, Table 1).

In the multivariate probability regression, most of these variables continued to be significantly related to HIO presence. Compared to markets with a low number of hospitals, markets with a moderate number of hospitals had a 19 percentage-point higher probability of HIO presence (*p* = 0.032) and markets with a high number of hospitals had a 19 percentage-point higher probability of HIO presence (*p* = 0.006, Table 2). Compared to non-competitive hospital markets, highly-competitive hospital markets had 25 percentage-point lower probability of HIO presence (*p* = 0.003, Table 2). Moderately-competitive hospital markets had a 12 percentage-point lower probability of HIO presence, though this effect was not statistically significant (*p* = 0.058, Table 2). Markets in the top quartile of for-profit hospital marketshare had a 7 percentage-point lower probability of HIO presence, though this effect was also not statistically significant (*p* = 0.163, Table 2).

**Table 2:** HIO Presence and Hospital Participation in Markets with different Hospital and Vendor Characteristics

Variables	Linear Probability Model Coefficients for HIO Presence		Linear Regression Coefficients for Hospital Participation	
Constant	−0.232	(0.158)	54.8**	(17.4)
Hospital Dynamics				
Number of Hospitals (Ref: Low 1–4)				
Moderate (5–8)	0.185*	(0.079)	−5.2	(5.8)
High (9+)	0.190**	(0.068)	−10.9*	(4.7)
Hospital Competition (Ref: Non-competitive, HHI= 0.46–1.00)				
Moderately Competitive (0.25–0.45)	− 0.118	(0.063)	−8.4	(4.7)
Highly Competitive (0.00–0.24)	−0.252**	(0.078)	−10.2	(6.0)
For-Profit Hospital Market Share (Ref: Low 0–57%)				
High For-Profit Hospital Market Share (> 57%)	−0.067	(0.053)	−5.6	(3.9)
EHR Vendor Dynamics				
Number of EHR Vendors (Ref: Low 1–2)				
Moderate (3–4)	0.208**	(0.070)	5.4	(5.0)
High (5+)	0.318***	(0.095)	7.0	(6.6)
Vendor Competition (Ref: Non-competitive, HHI= 0.63–1.00)				
Moderately Competitive (0.38–0.62)	0.033	(0.059)	−6.9	(4.5)
Highly Competitive (0.00–0.37)	−0.039	(0.072)	−11.2*	(5.6)
Alternative HIE Approach (Ref: No)				
Yes (50–100% of hospitals on Epic)	0.144**	(0.046)	1.2	(3.1)
Community Controls				
% Hospital Participation in Patient Centered Medical Home and/or Accountable Care Organizations	0.004***	(0.001)	0.1	(0.21)
Avg. % Revenue from Shared Risk Programs	0.004	(0.005)	0.1	(0.3)
% Inpatient Days Medicare	0.002	(0.002)	0.1	(0.2)
% Inpatient Days Medicaid	0.007	(0.004)	0.1	(0.3)
Hospital Beds per 1000 residents	−0.001	(0.002)	0.1	(0.2)
FTE Hospital Staff per 1000 residents	0.000	(0.000)	0.0	(0.0)
Percentage of Hospitals in Urban Settings	0.005***	(0.001)	0.0	(0.1)
Number of Physicians (Weighted County Average)	−0.000*	(0.000)	0.0	(0.0)
State Fixed Effects	Included		Included	
n	469		298	
R^2^	0.44		0.16	

Note: * *p*<0.05, ** *p*<0.01, *** *p*<0.001. All models include state fixed effects. Robust standard error in parentheses

Markets with more EHR vendors were more likely to have HIO presence. Compared to markets with a low number of vendors, markets with a moderate number of vendors had a 21 percentage-point higher probability of HIO presence (*p* = 0.002) while markets with a high number of vendors had a 32 percentage-point higher probability of HIO presence (*p* = 0.001, Table 2). Vendor competition did not have a statistically significant relationship with probability of HIO presence. Finally, having an alternative HIE approach was associated with a 14 percentage-point higher probability of HIO presence (p = 0.001, Table 2).

### Hospital participation in HIO

Bivariate analyses comparing hospital participation by market characteristics in the 298 markets with HIO presence revealed that markets with a higher level of HIO participation were more likely to have: (1) a low number of hospitals (*p* < 0.001), (2) low for-profit hospital marketshare (*p* < 0.001), (3) a low number of EHR vendors (*p* < 0.001), and (4) have an alternative HIE approach (*p* < 0.001, Table 1).

In multivariate regression analysis, two independent variables were significantly associated with hospital participation in HIOs. Compared to markets with a low number of hospitals, markets with a high number of hospitals had on average 11 percentage-points lower hospital participation (*p* = 0.023, Table 2). Though not statistically significant, markets with a moderate number of hospitals had on average 5 percentage-points lower hospital participation (*p* = 0.350, Table 2). Compared to markets with low vendor competition, markets with high vendor competition had on average 11 percentage-points lower hospital participation (*p* = 0.043, Table 2). Though not statistically significant, markets with moderate vendor competition had 7 percentage-points lower hospital participation (*p* = 0.151, Table 2).

### Robustness tests

Odds ratio estimates from the logistic regression model were similar to estimates from the linear probability model in both relative magnitude and significance (Additional file 2: Logistic Regression Model for HIO PResence in Markets with Different Hospital and Vendor Characteristics). The result of the robustness tests for multicollinearity showed that using continuous variables resulted in coefficients in the same direction as in the primary models (Additional file 3: Continuous Variable Models for HIO Presence and Level of Participation in HIOs). Excluding collinear variables did not substantially change standard errors (Additional file 4 and Additional file 5: Robustness Tests for Multicolinearity). While statistical significance did change in some cases, these changes suggest that the collinear variables are picking up the effect of the excluded variables, resulting in either significance if the excluded variable had an effect in the same direction of the collinear variable, or insignificance if the excluded variable had an effect in the opposite direction. These results suggest that the overall conclusions still hold.

## Discussion

This study examined the relationship between hospital and EHR vendor market dynamics, and HIO presence and hospital participation. We found that both hospital and EHR vendor dynamics (number of hospitals, hospital competition, number of vendors, and having an alternative HIE approach) are associated with HIO presence, while a greater number of hospitals and high vendor competition predicts lower hospital participation. These results supported some of our hypothesized relationships and contradicted others.

Consistent with what we predicted, markets with more hospitals and vendors were more likely to have an HIO. This suggests that markets with many hospitals or vendors may perceive a greater need for an HIO, leading to a greater likelihood of HIO presence. However, also as we predicted, competitive dynamics among hospitals appear to work against HIO presence. More competitive hospital markets were less likely to have an HIO. Contrary to what we predicted, vendor dynamics did not additionally limit HIO presence. Markets with an alternative, EHR vendor-led approach to HIE were more likely to have an HIO (and vendor competition was unrelated). While this finding contradicts our hypothesis that hospitals in markets with an alternative approach may be less inclined to support an HIO because the majority of hospitals can use Epic’s Care Everywhere platform to engage in HIE, a possible explanation is that this measure is serving as a proxy measure for health IT market maturity, which we would expect to increase the likelihood of having an HIO.

Our results on the level of hospital participation in markets with HIOs similarly pointed to the influence of hospital dynamics. Markets with more hospitals had lower HIO participation, which was surprising given our expectation that more hospitals would lead to greater HIO participation. It may be that hospital perception of HIO value is a function of the percent of hospitals in the market participating in the HIO. That is, HIOs in larger markets may have a harder time recruiting hospitals to participate since it takes many more participating hospitals to achieve the same value. Greater vendor competition was also associated with lower HIO participation, which was consistent with our prediction and suggests that competitive dynamics among vendors may impact how easy they make it for hospitals to connect to an HIO. Both findings point to the ongoing challenges facing HIO viability.

Our study contributes to the growing literature on HIO development and sustainability. While studies have found that hospital participation in health information exchange is growing [19], they do not identify how market dynamics may be influencing the *types* of exchanges that are developing. The types of health information exchange efforts in a market have important consequences for whether information exchange serves to enhance patient care or strengthen strategic relationships between providers [20]. Our study is consistent with prior work that has found that hospitals in highly competitive markets are less likely to participate in HIOs [7, 8, 16, 19]. However, by examining EHR vendor dynamics as well as market-level hospital dynamics as potential inhibitors of HIO presence, we meaningfully extend these findings by revealing that hospital competition is a potential inhibitor of HIO *presence* in a market, while EHR vendor competition may subsequently inhibit hospital participation in HIOs. An investigation of the effect of EHR vendor dynamics on HIOs is important to our understanding of HIO development and sustainability as EHR vendors are key stakeholders in HIE efforts and have the potential to make or break efforts to achieve community-level information exchange.

Our work has important policy implications. Through the CMS Meaningful Use Program and HITECH funding for HIOs, policymakers have tried to create conditions under which HIOs can become established and achieve broad provider participation. Recent concerns about their viability have primarily stemmed from vendor dynamics – including information blocking [6] and proprietary HIE networks [21]. In this context, we find that both hospital dynamics and vendor dynamics appear to be inhibiting HIO viability. It could be that competition between hospitals inhibits the formation of HIOs, while in areas with HIO presence, vendor competition gives rise to information blocking behaviors that create barriers to high hospital participation.

In the interim, our results suggest that efforts to-date have not created sufficient demand for geographically-based HIE. Policymakers may therefore be best off targeting market dynamics in their efforts to promote HIOs. For example, our results suggest that payment reform efforts (e.g. PCMH and ACO models) that incentivize hospitals to take on greater risk for the cost and quality of care that occurs in the market at large may introduce needed incentives for hospitals to participate in HIOs. Under these models, hospital participation in HIOs may improve the quality of care transitions, reducing readmission rates and subsequent penalties. It will be important to study these mechanisms directly as well as more broadly monitor changes in HIO activity as these policies progress and mature.

### Limitations

Our study has several limitations. First, missing data from the AHA IT supplement and AHA survey resulted in dropping 72 markets. These markets had fewer HSAs, lower hospital participation in PCMH and/or ACO models, fewer hospital beds, fewer employees, fewer physicians, fewer hospitals, were less urban, were more likely to be non-competitive hospital markets, and have a higher for-profit marketshare than the markets that were not dropped (Additional file 6: Descriptive Statistics for Areas in Sample and those Dropped from the Sample), which could limit generalizability of our results. Second, we were unable to determine whether hospitals in a given market participated in the same or different HIOs. Without this information, our analysis is unable to examine the possible confounding effect of HIO competition on HIO presence and HIO participation. Lastly, the analyses are associative and should not be used to draw conclusions about causality. Relatedly, we are unable to say whether our key independent variables contribute to HIO *formation*, or whether our key independent variables prevent HIO *closures*. There could also be *interactions* between hospital and vendor market dynamics that our measures failed to capture. Future research should examine these as well as the impact of ambulatory provider dynamics on HIO activity [22], since a major function of HIE is to facilitate information exchange with ambulatory providers.

## Conclusions

Market dynamics related to hospital competition appear to be the key factor impeding HIO presence while vendor competition may be a key factor limiting hospital participation in HIOs. Taken together these results suggest that efforts to improve HIO sustainability may need to address possible information blocking behaviors stemming from competitive vendor dynamics and make HIO participation more appealing to hospitals in competitive, larger markets, which may be best facilitated by expanding payment reform efforts and strengthening market-level incentives for valuable activities that HIOs enable.

## Additional files

Additional file 1: Conceptual Model. Conceptual model illustrating relationship between hospital and EHR vendor market dynamics, costs and benefits of HIO, perceived value of HIO, HIO presence, and level of participation in HIO. (DOCX 71 kb)

Additional file 2: Logistic Regression Model for HIO Presence in Markets with different Hospital and Vendor Characteristics. Sensitivity Analysis Results from Logistic Regression Model. (DOCX 172 kb)

Additional file 3: Continuous Variable Models for HIO Presence and Level of Participation in HIOs. Sensitivity Analysis Results from Continuous Variable Models (DOCX 93 kb)

Additional file 4: Robustness Test for Multicollinearity, Linear Probability Model. Sensitivity Analysis Results from Probability Models testing for Multicollinearity. (DOCX 109 kb)

Additional file 5: Robustness Test for Multicollinearity, Coefficients for Linear Regression Model. Sensitivity Analysis Results from Linear Regression Models testing for Multicollinearity. (DOCX 104 kb)

Additional file 6: Descriptive Statistics for Areas in Sample and those Dropped from the Sample. Descriptive Statistics for In and Out of Sample Observations. (DOCX 76 kb)

## Abbreviations

ACO: Accountable Care Organization
AHA: American Hospital Association
ANOVA: Analysis of Variance
CMS: Centers for Medicare & Medicaid Services
EHR: Electronic Health Record
HHI: Herfindahl-Hirschman Index
HIE: Health Information Exchange
HIO: Health Information Organization
HITECH: The Health Information Technology for Economic and Clinical Health (HITECH) Act
HRR: Health Referral Region
HSA: Health Service Area
OLS: Ordinary Least Squares
PCMH: Patient Centered Medical Home
