# Supplementary material for: The relationship between hospital and ehr vendor market dynamics on health information organization presence and participation
Source: BMC Med Inform Decis Mak. 2018 May 8;18:28. doi: 10.1186/s12911-018-0605-y (PMC5941339; doi:10.1186/s12911-018-0605-y)
Supplement: Supplementary file 1 — Conceptual Model. Conceptual model illustrating relationship between hospital and EHR vendor market dynamics, costs and benefits of HIO, perceived value of HIO, HIO presence, and level of participation in HIO. (DOCX 71 kb) [file 12911_2018_605_MOESM1_ESM.docx]

Additional file 1. Conceptual Model
