# Supplementary material for: The relationship between hospital and ehr vendor market dynamics on health information organization presence and participation
Source: BMC Med Inform Decis Mak. 2018 May 8;18:28. doi: 10.1186/s12911-018-0605-y (PMC5941339; doi:10.1186/s12911-018-0605-y)
Supplement: Supplementary file 2 — Logistic Regression Model for HIO Presence in Markets with different Hospital and Vendor Characteristics. Sensitivity Analysis Results from Logistic Regression Model. (DOCX 172 kb) [file 12911_2018_605_MOESM2_ESM.docx]

Additional file 2. Logistic Regression Model for HIO Presence in Markets with different Hospital and Vendor Characteristics

| Variables | Odds Ratios for HIO Presence | | Percentage Change in Probability given unit change in IV | |  |
| --- | --- | --- | --- | --- | --- |
| Constant |  |  |  |  |  |
| ***Hospital and Vendor Dynamics*** |  |  |  |  |  |
| Number of Hospitals (Ref: Low 1-4) |  |  |  |  |  |
| Moderate (5-8) | 2.317 | (1.489) | 0.306 | (0.235) |  |
| High (9+) | 3.409** | (1.470) | 0.446** | (0.159) |  |
| Hospital Competition (Ref: Non-competitive 0.46-1.00) |  |  |  |  |  |
| Moderately Competitive (0.25-0.45) | | 0.383* | (0.162) | -0.349* | (0.155) |
| Highly Competitive (0.00-0.24) | | 0.134** | (0.085) | -0.733** | (0.235) |
| For-Profit Market Share (Ref: 0-27%) |  |  |  |  |  |
| High marketshare (27%+) | 0.677 | (0.253) | -0.142 | (0.136) |  |
| Number of EHR Vendors (Ref: Low 1-2) |  |  |  |  |  |
| Moderate (3-4) | 4.808** | (2.303) | 0.572** | (0.178) |  |
| High (5+) | 15.140*** | (11.261) | 0.989*** | (0.277) |  |
| Vendor Competition (Ref: Non-competitive 0.63-1.00) |  |  |  |  |  |
| Moderately Competitive (0.38-0.62) | 0.996 | (0.404) | -0.001 | (0.148) |  |
| Highly Competitive (0.00-0.37) | 0.488 | (0.293) | -0.262 | (0.220) |  |
| Alternative HIE Approach (Ref: No) |  |  |  |  |  |
| Yes (50-100% of hospitals on Epic) | 2.542** | (0.835) | 0.340** | (0.121) |  |
| ***Community Controls*** |  |  |  |  |  |
| % Hospital Participation in Patient Centered Medical Home and/or Accountable Care Organizations | 1.029*** | (0.007) | 0.010*** | (0.003) |  |
| Avg. % Revenue from Shared Risk Programs | 1.018 | (0.041) | 0.007 | (0.015) |  |
| % Inpatient Days Medicare | 1.020 | (0.018) | 0.007 | (0.006) |  |
| % Inpatient Days Medicaid | 1.061* | (0.026) | 0.022* | (0.009) |  |
| Hospital Beds per 1000 residents | 0.945 | (0.033) | -0.021 | (0.013) |  |
| FTE Hospital Staff per 1000 residents | 1.012 | (0.007) | 0.004 | (0.002) |  |
| Percentage of Hospitals in Urban Settings | 1.037*** | (0.006) | 0.013*** | (0.002) |  |
| Number of Physicians (Weighted County Average) | 1.000* | (0.000) | -0.000* | (0.000) |  |
| State Fixed Effects | Included |  |  |  |  |
| n | 460 |  |  |  |  |
| Prob > chi2 | <0.001 |  |  |  |  |
|  |  |  |  |  |  |

Standard errors in parentheses, Legend: *p <0.05, **p <0.01, *** p< 0.001

^1^8 markets in 5 states were dropped due to perfect prediction
