# Supplementary material for: The relationship between hospital and ehr vendor market dynamics on health information organization presence and participation
Source: BMC Med Inform Decis Mak. 2018 May 8;18:28. doi: 10.1186/s12911-018-0605-y (PMC5941339; doi:10.1186/s12911-018-0605-y)
Supplement: Supplementary file 3 — Continuous Variable Models for HIO Presence and Level of Participation in HIOs. Sensitivity Analysis Results from Continuous Variable Models (DOCX 93 kb) [file 12911_2018_605_MOESM3_ESM.docx]

Additional file 3. Continuous Variable Models for HIO Presence and Level of Participation in HIOs

| Variables | Linear Probability Model Coefficients for HIO Presence | | Linear Regression Coefficients for Hospital Participation | |
| --- | --- | --- | --- | --- |
| Constant | -0.180 | (0.180) | 23.03 | (18.42) |
| ***Hospital Dynamics*** |  |  |  |  |
| Number of Hospitals | 0.014 | (0.009) | -0.28 | (0.39) |
| Hospital Herfindahl-Hirschman Index (HHI) | 0.188 | (0.138) | 30.91* | (14.03) |
| For-Profit Market Share | -0.001 | (0.001) | -0.01 | (0.98) |
| ***EHR Vendor Dynamics*** |  |  |  |  |
| Number of EHR Vendors | 0.040* | (0.017) | -0.01 | (0.98) |
| Vendor Herfindahl-Hirschman Index (HHI) | -0.239 | (0.133) | 6.88 | (10.96) |
| Alternative HIE Approach (Ref: No) |  |  |  |  |
| Yes (50-100% of hospitals on Epic) | 0.174*** | (0.046) | 2.30 | (3.68) |
| ***Community Controls*** |  |  |  |  |
| % Hospital Participation in Patient Centered Medical Home and/or Accountable Care Organizations | 0.004*** | (0.001) | 0.09 | (0.09) |
| Avg. % Revenue from Shared Risk Programs | 0.004 | (0.005) | 0.04 | (0.36) |
| % Inpatient Days Medicare | 0.003 | (0.002) | 0.11 | (0.21) |
| % Inpatient Days Medicaid | 0.007 | (0.004) | 0.08 | (0.31) |
| Hospital Beds per 1000 residents | -0.002 | (0.003) | 0.09 | (0.11) |
| FTE Hospital Staff per 1000 residents | -0.000 | (0.001) | 0.01 | (0.03) |
| Percentage of Hospitals in Urban Settings | 0.005*** | (0.001) | 0.00 | (0.08) |
| Number of Physicians (Weighted County Average) | -0.000* | (0.000) | -0.00 | (0.00) |
| State Fixed Effects | Included |  | Included |  |
| n | 469 |  | 298 |  |

Standard errors in parentheses, Legend: *p <0.05, **p <0.01, *** p< 0.001
