# Supplementary material for: The relationship between hospital and ehr vendor market dynamics on health information organization presence and participation
Source: BMC Med Inform Decis Mak. 2018 May 8;18:28. doi: 10.1186/s12911-018-0605-y (PMC5941339; doi:10.1186/s12911-018-0605-y)
Supplement: Supplementary file 4 — Robustness Test for Multicollinearity, Linear Probability Model. Sensitivity Analysis Results from Probability Models testing for Multicollinearity. (DOCX 109 kb) [file 12911_2018_605_MOESM4_ESM.docx]

Additional file 4. Robustness Test for Multicollinearity, Linear Probability Model

|  | ***(1)*** | ***(2)*** | | ***(3)*** | | ***(4)*** | | ***(5)*** |  |
| --- | --- | --- | --- | --- | --- | --- | --- | --- | --- |
|  | ***Original Model*** | ***Excluding Number of Hospitals*** | | ***Excluding Number of Vendors*** | | ***Excluding Hospital competition*** | | ***Excluding Vendor competition*** |  |
| Constant | -0.23 (0.16) | -0.27 (0.16) | -0.19 (0.16) | | -0.29 (0.16) | | -0.24 (0.16) | | |
| ***Hospital Dynamics*** |  |  |  | |  | |  | | |
| Number of Hospitals (Ref: Low 1-4) |  |  |  | |  | |  | | |
| Moderate (5-8) | 0.19** (0.07) |  | 0.27*** (0.06) | | 0.14* (0.07) | | 0.19** (0.07) | | |
| High (9+) | 0.19* (0.08) |  | 0.32*** (0.07) | | 0.09 (0.08) | | 0.19* (0.08) | | |
| Hospital Competition (Ref: Non-competitive 0.46-1.00) |  |  |  | |  | |  | | |
| Moderately Competitive (0.25-0.45) | -0.12 (0.06) | -0.08 (0.06) | -0.10 (0.06) | |  | | -0.11 (0.06) | | |
| Highly Competitive (0.00-0.24) | -0.25** (0.08) | -0.18* (0.07) | -0.22** (0.08) | |  | | -0.25*** (0.08) | | |
| For-Profit Market Share (Ref: 0-27%) |  |  |  | |  | |  | | |
| High marketshare (27%+) | -0.07 (0.05) | -0.07 (0.05) | -0.07 (0.05) | | -0.06 (0.05) | | -0.07 (0.05) | | |
| ***EHR Vendor Dynamics*** |  |  |  | |  | |  | | |
| Number of EHR Vendors (Ref: Low 1-2) |  |  |  | |  | |  | | |
| Moderate (3-4) | 0.21** (0.07) | 0.29*** (0.06) |  | | 0.19** (0.07) | | 0.20** (0.06) | | |
| High (5+) | 0.32*** (0.09) | 0.41*** (0.08) |  | | 0.29** (0.10) | | 0.28*** (0.08) | | |
| Vendor Competition (Ref: Non-competitive 0.63-1.00) |  |  |  | |  | |  | | |
| Moderately Competitive (0.38-0.62) | 0.03 (0.06) | 0.04 (0.06) | 0.08 (0.06) | | -0.02 (0.06) | |  | | |
| Highly Competitive (0.00-0.37) | -0.04 (0.07) | -0.04 (0.07) | 0.09 (0.06) | | -0.11 (0.07) | |  | | |
| Alternative HIE Approach (Ref: No) |  |  |  | |  | |  | | |
| Yes (50-100% of hospitals on Epic) | 0.14** (0.05) | 0.16*** (0.05) | 0.16*** (0.05) | | 0.16*** (0.05) | | 0.15** (0.05) | | |
| ***Community Controls*** |  |  |  | |  | |  | | |
| % Hospital Participation in Patient Centered Medical Home and/or Accountable Care Organizations | 0.004*** (0.001) | 0.004*** (0.001) | 0.004*** (0.001) | | 0.004*** (0.001) | | 0.004*** (0.001) | | |
| Avg. % Revenue from Shared Risk Programs | 0.00 (0.00) | 0.01 (0.00) | 0.00 (0.00) | | 0.00 (0.00) | | 0.00 (0.00) | | |
| % Inpatient Days Medicare | 0.00 (0.00) | 0.00 (0.00) | 0.00 (0.00) | | 0.00 (0.00) | | 0.00 (0.00) | | |
| % Inpatient Days Medicaid | 0.01 (0.00) | 0.01 (0.00) | 0.01 (0.00) | | 0.01 (0.00) | | 0.01* (0.00) | | |
| Hospital Beds per 1000 residents | -0.00 (0.00) | -0.00 (0.00) | -0.00 (0.00) | | -0.00 (0.00) | | -0.00 (0.00) | | |
| FTE Hospital Staff per 1000 residents | 0.00 (0.00) | 0.00 (0.00) | 0.00 (0.00) | | 0.00 (0.00) | | 0.00 (0.00) | | |
| Percentage of Hospitals in Urban Settings | 0.01*** (0.00) | 0.01*** (0.00) | 0.01*** (0.00) | | 0.01*** (0.00) | | 0.01*** (0.00) | | |
| Number of Physicians (Weighted County Average) | -0.00* (0.00) | -0.00* (0.00) | -0.00* (0.00) | | -0.00* (0.00) | | -0.00* (0.00) | | |
| N | 469 | 469 | | 469 | | 469 | | 469 |  |

Robust standard Errors in parentheses, Legend: *p <0.05, **p <0.01, *** p< 0.001; state fixed effects included in all models
