# Supplementary material for: The relationship between hospital and ehr vendor market dynamics on health information organization presence and participation
Source: BMC Med Inform Decis Mak. 2018 May 8;18:28. doi: 10.1186/s12911-018-0605-y (PMC5941339; doi:10.1186/s12911-018-0605-y)
Supplement: Supplementary file 5 — Robustness Test for Multicollinearity, Coefficients for Linear Regression Model. Sensitivity Analysis Results from Linear Regression Models testing for Multicollinearity. (DOCX 104 kb) [file 12911_2018_605_MOESM5_ESM.docx]

Additional file 5. Robustness Test for Multicollinearity, Coefficients for Linear Regression Model

|  | ***(1)*** | ***(2)*** | ***(3)*** | ***(4)*** | ***(5)*** |
| --- | --- | --- | --- | --- | --- |
|  | ***Original Model*** | ***Excluding Number of Hospitals*** | ***Excluding Number of Vendors*** | ***Excluding Hospital competition*** | ***Excluding Vendor competition*** |
| Constant | 54.77** (17.45) | 51.56** (17.60) | 57.42** (17.26) | 51.77** (17.49) | 51.50** (17.42) |
| ***Hospital Dynamics*** |  |  |  |  |  |
| Number of Hospitals (Ref: Low 1-4) |  |  |  |  |  |
| Moderate (5-8) | -10.89* (4.70) |  | -8.82* (4.29) | -13.27** (4.52) | -10.14* (4.70) |
| High (9+) | -5.19 (5.76) |  | -2.36 (5.18) | -7.92 (5.37) | -4.31 (5.75) |
| Hospital Competition (Ref: Non-competitive 0.46-1.00) |  |  |  |  |  |
| Moderately Competitive (0.25-0.45) | -8.41 (4.74) | -10.95* (4.66) | -7.58 (4.68) |  | -11.35* (4.39) |
| Highly Competitive (0.00-0.24) | -10.17 (5.96) | -12.74* (5.63) | -9.04 (5.87) |  | -14.06* (5.57) |
| For-Profit Market Share (Ref: 0-27%) |  |  |  |  |  |
| High marketshare (27%+) | -5.56 (3.90) |  | -5.89 (3.88) |  | -6.56 (3.87) |
| ***EHR Vendor Dynamics*** |  |  |  |  |  |
| Number of EHR Vendors (Ref: Low 1-2) |  |  |  |  |  |
| Moderate (3-4) | 5.37 (4.96) | 2.28 (4.52) |  | 4.50 (4.91) | 2.18 (4.72) |
| High (5+) | 7.03 (6.62) | 4.83 (6.06) |  | 5.54 (6.56) | 1.49 (5.75) |
| Vendor Competition (Ref: Non-competitive 0.63-1.00) |  |  |  |  |  |
| Moderately Competitive (0.38-0.62) | -11.24* (5.64) | -11.49* (5.65) | -8.66 (5.07) | -15.71** (5.31) |  |
| Highly Competitive (0.00-0.37) | -6.93 (4.51) | -6.82 (4.49) | -5.88 (4.38) | -11.32** (4.09) |  |
| Alternative HIE Approach (Ref: No) |  |  |  |  |  |
| Yes (50-100% of hospitals on Epic) | 1.18 (3.11) | 1.47 (3.11) | 1.63 (3.08) | 1.88 (3.10) | 1.19 (3.12) |
| ***Community Controls*** |  |  |  |  |  |
| % Hospital Participation in Patient Centered Medical Home and/or Accountable Care Organizations | 0.09 (0.07) | 0.10 (0.07) | 0.08 (0.07) | 0.10 (0.07) | 0.09 (0.07) |
| Avg. % Revenue from Shared Risk Programs | 0.09 (0.32) | 0.01 (0.32) | 0.04 (0.31) | 0.10 (0.32) | 0.09 (0.32) |
| % Inpatient Days Medicare | 0.10 (0.21) | 0.08 (0.21) | 0.07 (0.21) | 0.12 (0.21) | 0.13 (0.21) |
| % Inpatient Days Medicaid | 0.08 (0.26) | 0.10 (0.26) | 0.07 (0.26) | 0.12 (0.26) | 0.11 (0.26) |
| Hospital Beds per 1000 residents | 0.10 (0.17) | 0.05 (0.18) | 0.09 (0.17) | 0.09 (0.17) | 0.10 (0.18) |
| FTE Hospital Staff per 1000 residents | -0.02 (0.03) | -0.01 (0.03) | -0.02 (0.03) | -0.02 (0.03) | -0.02 (0.03) |
| Proportion of Hospitals in Urban Settings | 0.00 (0.06) | -0.00 (0.06) | -0.01 (0.06) | -0.02 (0.06) | -0.00 (0.06) |
| Number of Physicians (Weighted County Average) | -0.00 (0.00) | -0.00 (0.00) | -0.00 (0.00) | -0.00 (0.00) | -0.00 (0.00) |
| State Fixed Effects | Included | Included | Included | Included | Included |
| N | 298 | 298 | 298 | 298 | 298 |

Legend: *p <0.05, **p <0.01, ***p<0.001
