# Supplementary material for: The relationship between hospital and ehr vendor market dynamics on health information organization presence and participation
Source: BMC Med Inform Decis Mak. 2018 May 8;18:28. doi: 10.1186/s12911-018-0605-y (PMC5941339; doi:10.1186/s12911-018-0605-y)
Supplement: Supplementary file 6 — Descriptive Statistics for Areas in Sample and those Dropped from the Sample. Descriptive Statistics for In and Out of Sample Observations. (DOCX 76 kb) [file 12911_2018_605_MOESM6_ESM.docx]

Additional file 6. Descriptive Statistics for Areas in Sample and those Dropped from the Sample

| Variables | Sample | | Dropped from Sample | |
| --- | --- | --- | --- | --- |
| Number of Areas | 469 |  | 72 |  |
| ***Continuous Variables*** | ***Mean*** | ***Std Dev.*** | ***Mean*** | ***Std Dev.*** |
| Number of HSAs* | 5.64 | (5.70) | 2.56 | (2.46) |
| % Hospital Participation in PCMH and/or ACO* | 27.55 | (26.56) | 19.94 | (33.85) |
| % Inpatient Days Medicare | 51.91 | (11.67) | 54.75 | (18.17) |
| % Inpatient Days Medicaid | 18.73 | (8.65) | 20.39 | (17.79) |
| Hospital Beds per 1000 residents* | 23.84 | (30.75) | 7.63 | (8.63) |
| FTE Hospital Staff per 1000 residents* | 163.96 | (196.96) | 53.80 | (61.82) |
| Percentage of Hospitals in Urban Settings* | 62.42 | (35.38) | 51.32 | (43.46) |
| Number of Physicians (Weighted County Average)* | 409.25 | (769.24) | 297.40 | (721.98) |
|  |  |  |  |  |
| ***Categorical Variables*** | ***N*** | ***Percent*** | ***N*** | ***Percent*** |
| Hospital Competition* |  |  |  |  |
| Non-Competitive (0.46-1.00) | 127 | 27% | 53 | 74% |
| Moderately Competitive (0.25-0.45) | 166 | 35% | 14 | 19% |
| Highly Competitive (0.00-0.24) | 176 | 38% | 5 | 7% |
|  |  |  |  |  |
| Number of Hospitals* |  |  |  |  |
| Low (1-4) | 175 | 37% | 57 | 79% |
| Moderate (5-8) | 128 | 27% | 12 | 17% |
| High (9+) | 166 | 35% | 3 | 4% |
| For-profit Marketshare |  |  |  |  |
| Low marketshare (0-27%) | 357 | 76% | 50 | 68% |
| High marketshare (27%+) | 112 | 24% | 23 | 32% |

*Difference between groups is statistically significantly different from 0 at p<0.05
